# The Cytotoxic Effects of Fine Particulate Matter (PM_2.5_) from Different Sources at the Air–Liquid Interface Exposure on A549 Cells

**DOI:** 10.3390/toxics12010021

**Published:** 2023-12-25

**Authors:** Zhansheng Yan, Pengxiang Ge, Zhenyu Lu, Xiaoming Liu, Maoyu Cao, Wankang Chen, Mindong Chen

**Affiliations:** 1 Collaborative Innovation Center of Atmospheric Environment and Equipment Technology, Jiangsu Key Laboratory of Atmospheric Environment Monitoring and Pollution Control, School of Environmental Science and Engineering, Nanjing University of Information Science & Technology, Nanjing 210044, China; 20211248092@nuist.edu.cn (Z.Y.); gepx@nuist.edu.cn (P.G.); l17864182144@163.com (X.L.); 20211248056@nuist.edu.cn (W.C.); 2 School of Atmospheric Sciences, Nanjing University, Nanjing 210023, China; 20201248092@nuist.edu.cn

**Keywords:** PM_2.5_, air–liquid interface exposure, cell toxicity, oxidative stress

## Abstract

The health of humans has been negatively impacted by PM_2.5_ exposure, but the chemical composition and toxicity of PM_2.5_ might vary depending on its source. To investigate the toxic effects of particulate matter from different sources on lung epithelial cells (A549), PM_2.5_ samples were collected from residential, industrial, and transportation areas in Nanjing, China. The chemical composition of PM_2.5_ was analyzed, and toxicological experiments were conducted. The A549 cells were exposed using an air–liquid interface (ALI) exposure system, and the cytotoxic indicators of the cells were detected. The research results indicated that acute exposure to different sources of particulate matter at the air–liquid interface caused damage to the cells, induced the production of ROS, caused apoptosis, inflammatory damage, and DNA damage, with a dose–effect relationship. The content of heavy metals and PAHs in PM_2.5_ from the traffic source was relatively high, and the toxic effect of the traffic–source samples on the cells was higher than that of the industrial– and residential–source samples. The cytotoxicity of particulate matter was mostly associated with water–soluble ions, carbon components, heavy metals, PAHs, and endotoxin, based on the analysis of the Pearson correlation. Oxidative stress played an important role in PM_2.5_–induced biological toxicity.

## 1. Introduction

With the rapid development of industrialization and urbanization, the impact of atmospheric pollution on human health has been increasingly significant [1]. The International Agency for Research on Cancer (IARC) has classified fine particulate matter (<2.5 μm, PM_2.5_) as a Class I carcinogen. PM_2.5_ was a major air pollutant that was produced by traffic exhausts, industrial emissions, burning coal and fuel, and other human activities, as well as aerosol formation in natural processes [2,3,4]. PM_2.5_ could be suspended in the air for a long time and carry various harmful substances (such as polycyclic aromatic hydrocarbons, heavy metals, etc.). PM_2.5_ could enter the deep respiratory system of the human body and be transported to the alveoli and blood, causing direct damage to the respiratory system and causing a series of diseases such as to the cardiovascular system through blood circulation [5,6,7].

PM_2.5_ was not a single chemical substance, but a complex mixture produced from multiple sources [8]. PM_2.5_ might contain different chemical components and pollutants due to its different sources and formation pathways, leading to differences in the health effects of particulate matter from different sources [9,10]. A study has shown that PM_2.5_ from urban, suburban, and traffic–affected areas in Tehran exhibited different levels of toxicity to A549 cells, while samples from urban locations exhibited higher levels of cytotoxicity [11]. Thus, it was essential to comprehend how PM_2.5_ from various sources affected human health and to research the potentially toxic features of PM_2.5_ from various sources.

Previous studies have shown that the chemical components (water–soluble ions, carbonaceous components, heavy metals, and polycyclic aromatic hydrocarbons) in PM_2.5_ were one of the key factors leading to its toxic effects on cells [12,13,14]. Water–soluble ions could lead to adverse effects such as cellular oxidative stress and inflammatory reactions [4,15]. Heavy metals, polycyclic aromatic hydrocarbons (PAHs), and their oxygen–containing derivatives could induce cytotoxicity, oxidative stress, inflammatory response, and DNA damage [15,16,17,18,19]. Oxidative stress was an important molecular mechanism for PM_2.5_–induced damage, and reactive oxygen species (ROS) –mediated oxidative stress was believed to play a crucial role in PM_2.5_–induced cytotoxicity [20,21,22]. Research has shown that ROS could induce oxidative stress damage, leading to DNA damage and cell apoptosis [12,21,23].

The air–liquid exposure device was a novelty experimental method for direct contact between air and cells at the air–liquid interface (ALI). Compared with traditional immersion exposure methods, the ALI exposure could better simulate the contact process between PM_2.5_ and cells, which was closer to the exposure mode of the human body in actual living environments and helped to more accurately simulate the impact of PM_2.5_ on the respiratory system [24]. In this study, we collected different particulate matter samples under the influence of residential areas, industry, and road traffic in Nanjing, and determined their composition. A549 cells were exposed at the ALI using the Vitrocell Cloud 12 system. Based on the evaluation of post–exposure cytotoxicity indicators, we explored the acute exposure toxicity and toxicity differences in PM_2.5_ from different sources on A549 cells. The correlation between the toxicity indicators and the chemical composition of particulate matter were analyzed, and the main toxic components of PM_2.5_ were explored.

## 2. Materials and Methods

### 2.1. PM_2.5_ Collection and Preparation

Three sampling points were selected for the collection of the PM_2.5_ samples, located at the entrance of the Nanjing Yangtze River Bridge (32°05′42′′ N, 118°45′21′′ E), the roof of the library of the Nanjing University of Information Science and Technology (32°12′8′′ N, 118°42′49′′ E), and the Nanjing Pukou Chemical Industrial Park (32°15′11′′ N, 118°46′23′′ E). The Nanjing Yangtze River Bridge was selected as the sampling site for collecting traffic–source PM_2.5_, with a sampling time of January 2016. The Nanjing University of Information Science and Technology was selected as the sampling site for collecting residential–source PM_2.5_, with a sampling time of February 2016. The Nanjing Pukou Chemical Industrial Park was selected as the sampling site for collecting industrial–source PM_2.5_, with a sampling time of March 2016. Each sampling point collected 25 samples, for a total of 75 samples. PM_2.5_ was collected on a quartz fiber filter through a high–flow sampler with a sampling flow rate of 1.13 m^3^/min, and samples were collected continuously for 24 h. Before sampling, the quartz fiber filter was baked at 450 °C for 6 h in a muffle furnace to remove organic matter and impurities from the filter. The filter was weighed both before and after sampling, and it was kept in a desiccator at room temperature for 24 h. These PM_2.5_ samples in the same source were cut into pieces, and ultrasonic extraction was performed 3 times with ultrapure water, each time for 20 min. To collect the PM_2.5_ samples, the solution was filtered through 8 layers of sterile gauze and then put in freeze–drying equipment to be vacuum freeze–dried. For later usage, the samples were kept in a refrigerator at –20 °C in the dark.

### 2.2. Analysis of PM_2.5_ Chemical Components

The primary water–soluble inorganic ions (Na^+^, NH_4_^+^, K^+^, Mg^2+^, Ca^2+^, F^−^, Cl^−^, SO_4_^2−^, and NO_3_^−^) were measured using multi-function ion chromatograph (IC, Dionex, Sunnyvale, CA, USA). The amount of elemental carbon (EC) and organic carbon (OC) was measured using an organic carbon analyzer (RT–4, Sunset Laboratory, Portland, OR, USA). For the analysis of PAHs, a certain amount of PM_2.5_ samples were dissolved in dichloromethane, ultrasonically extracted for 30 min, and repeated twice. After ultrasonic treatment, the samples were filtered using the 0.22μm PTFE filter. After filtration, nitrogen was blown to 200 μL for analysis. Gas chromatography–mass spectrometry (GC–MS, Agilent, Santa Clara, CA, USA) was used to determine the presence of PAHs. For the analysis of metal elements, a certain amount of PM_2.5_ samples were dissolved in 65% HNO_3_ after microwave digestion. The inductively coupled plasma mass spectrometer (ICP–MS, Thermo Fisher Scientific, Waltham, MA, USA) was used to assess the presence of 17 different heavy metals in the samples. The endotoxin detection kit (Beyotime, Shanghai, China) was used to detect the endotoxin content in the PM_2.5_ samples, which was based on the horseshoe crab reagent colorimetric method for detection. The experimental operation was carried out according to the instructions provided by the reagent kit. The microplate reader (Molecular Devices, San Jose, CA, USA) was used to measure the absorbance of the sample at a wavelength of 545 nm. The blank value was deducted from each result. These components’ recovery rate was within the desired range of 100 ± 15%, guaranteeing the data’s accuracy.

### 2.3. Cell Culture and Gas–Liquid Interface Exposure

A549 cells were provided by the stem cell bank of the Chinese Academy of Sciences. A549 cells were cultivated in RPMI–1640 media in an incubator set at 37 °C with 5% CO_2_ (Thermo Fisher Scientific, Waltham, MA, USA), supplemented with 10% fetal bovine serum (FBS, Bioagrio Science, Nanjing, China). The cell growth status was observed under the microscope, and cell passage culture was carried out when cell growth occupied 80–90% of the dish area. Cells were cultivated on 12–well Transwell culture plates (Labselect, Nanjing, China) with 50,000 cells per cell chamber for all exposure studies. In each cell chamber, 0.5 milliliters of RPMI-1640 medium was applied to the upper side and 1 milliliter to the lower. The surface area of the cell chamber was 1.12 cm^2^, and the pore size was 0.4μm polyester film. A549 cells were incubated under immersion conditions for 12 h to adhere to the wall and form a tightly adhered cell layer. After the upper layer of the culture medium was removed from the cell chamber, the cells were incubated under the air–liquid interface conditions for another 4 h until exposure experiments were conducted.

The ALI exposure of PM_2.5_ to A549 cells was achieved using a cloud system toxicology instrument (Vitrocell Cloud 12, Vitrocell Systems, Waldkirch, Germany). An amount of 4 mL of RPMI–1640 medium was added to each exposure module of the Vitrocell Cloud 12 system, making the level of medium slightly higher than the height of the PET membrane in the cell chamber to ensure sufficient contact between the medium and cells. The cloud system toxicology instrument has a warming system, which stabilizes the temperature at 37 °C throughout the exposure process, providing favorable living conditions for the cells. Four PM_2.5_ exposure gradients were set: 0 (control), 25 μg, 50 μg, and 100 μg. The PM_2.5_ samples were dissolved in phosphate–buffered saline (PBS), and treated with ultrasonic and vortexing, and the Transwell chamber was placed in the exposure module. The particulate exposure solution was atomized using an aerosolizer (Aeroneb Lab, Aerogen, Galway, Ireland), and the PM_2.5_ deposition mass on the chamber was 25, 50, and 100 μg for 5 min of continuous exposure. After the ALI exposure, the lower medium was replaced with 1 mL of RPMI–1640 medium without FBS, and the cell plate was placed in an incubator for 4 h to measure the cytotoxicity indicators [25,26]. Three parallel experiments were set up for the control group and exposure group.

### 2.4. Cell Vitality Detection

The CCK–8 kit (Beyotime, Shanghai, China) was used to determine the viability of A549 cells. WST–8 in the CCK–8 reagent reacted with intracellular dehydrogenases and was reduced to generate yellow WST–8 formazan. The color of the reaction solution was positively correlated with the number of viable cells. After exposure, 1 milliliter of RPMI–1640 medium containing 10% CCK–8 was added to the upper chamber of each cell chamber, and the culture plates were incubated in darkness at 37 °C for 2 h. An amount of 100 µL of CCK–8 solution was transferred from each cell chamber to a 96–well plate, with three repeated wells in each cell chamber. At 450 nm in wavelength, the optical density (OD) of the sample was measured with the microplate reader. The ratio of (OD _sample_ − OD _blank_)/(OD _control_ − OD _blank_) × 100% was used to express cell vitality.

### 2.5. ROS Detection

The DCFH–DA probe was used to detect the level of cellular ROS. Dimethyl sulfoxide (DMSO) was used to dissolve DCFH–DA powder (Sigma, St. Louis, MO, USA) to create a 10 mM stock solution. This was subsequently diluted to 10μM using RPMI–1640 medium. Each chamber added 500 μL of DCFH–DA solution, which was then incubated for 20 min at 37 °C in the dark. Each chamber was washed three times with PBS to thoroughly remove the extracellular DCFH–DA solution. Trypsin without EDTA was used to digest the cells. PBS was used to collect the cells, and the flow cytometry (CytoFLEX, Beckman Coulter, Pasadena, CA, USA) was used to detect the cells using an excitation wavelength of 488 nm and an emission wavelength of 525 nm. The ratio of the fluorescence intensity between the sample group and the control group was used to express the level of ROS.

### 2.6. Detection of Inflammatory Factors

With the aid of the enzyme–linked immunosorbent assay (ELISA), the amounts of pro-inflammatory cytokines were determined. Tumor necrosis factor (TNF–α) and interleukin–6 (IL–6) were inflammatory factors involved in particulate matter mediation. TNF–α was a pre–inflammatory response factor that could promote the secretion of inflammatory factor IL–6. The overexpression of IL–6 was associated with cellular inflammation [27,28,29]. Inflammatory factors (TNF–α and IL–6) were detected using an ELISA kit (Jiangsu Meimian Industrial Co., Ltd., Nanjing, China), and the absorbance was measured at a wavelength of 450 nm using a microplate reader. The levels of inflammatory factors in the supernatant were detected to determine the inflammatory damage effect of particulate matter on the cells. 

### 2.7. Detection of Cell Apoptosis Rate

The Annexin V–FITC/PI cell apoptosis detection kit (Beyotime, Shanghai, China) was used to determine the apoptosis rate of the A549 cells. The experimental operation was carried out according to the operating instructions provided by the reagent kit manufacturer. The flow cytometry was used to detect the cell apoptosis rates.

### 2.8. DNA Damage Detection

DNA damage was detected using the γ–H2AX immunofluorescence DNA damage detection kit (Beyotime, Shanghai, China). The experimental operation was carried out according to the operating instructions provided by the reagent kit manufacturer. The cells stained with fluorescence were observed and captured using the fluorescence microscope (Jiangnan NIB910, Yong Xin Corporation, Ningbo, China). γ–H2AX exhibited green fluorescence at an excitation wavelength of 488 nm. The Image J software was used to calculate the fluorescence intensity. The ratio of the sample group’s fluorescence intensity to that of the control group was used to express the degree of DNA damage.

### 2.9. Data Analysis

To guarantee that the results were accurate, each experiment was conducted three times. The mean ± standard deviation (SD) was used to express the experimental data. The statistical program SPSS (IBM Statistics SPSS 27.0) was used to conduct the analysis. One–way analysis of variance (ANOVA) was used to assess the differences between concentration groups, and Pearson correlation analysis was used to ascertain the relationship between PM_2.5_ components and cytotoxicity. In every experiment, the difference was statistically significant when the threshold of statistical testing was *p* < 0.05, and very significant when it was *p* < 0.01.

## 3. Results and Discussion

### 3.1. Analysis of PM_2.5_ Mass Concentration and Chemical Composition

**Figure 1:**
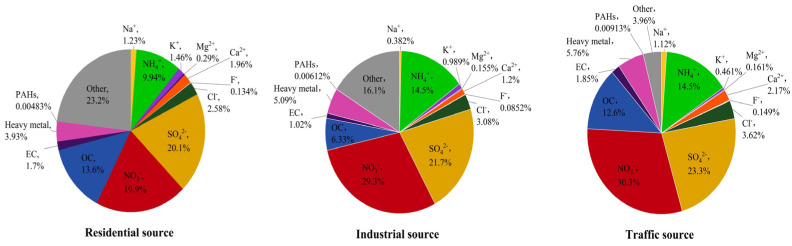
The mass concentration ratio of the main components of PM_2.5_ from different sources in Nanjing, China.

The average concentration of PM_2.5_ in different regions of Nanjing City was 111.32 ± 24.49 μg/m^3^ for the industrial sources, 82.37 ± 33.61 μg/m^3^ for the traffic sources, and 43.87 ± 16.30 μg/m^3^ for the residential sources, from high to low. The mass concentration ratios of the main components of PM_2.5_ from the different sources are summarized as follows (Figure 1). The average values of the PM_2.5_ components from the different sources are listed in the supplementary information (Tables S1–S5). From Figure 1, it can be seen that water–soluble ions accounted for 57.59%, 71.39%, and 75.78% of the PM_2.5_ mass in the residential, industrial, and traffic sources, respectively, making them the largest contributors to the PM_2.5_ mass concentration. The NO_3_^−^/SO_4_^2−^ ratio was often used to determine the main source of atmospheric particulate pollution. When the ratio was larger than 1, it meant that mobile sources, like car exhausts, dominated the emission source; when it was less than 1, it meant that stationary sources, such as coal combustion, dominated [4,30]. The NO_3_^−^/SO_4_^2−^ ratios of the PM_2.5_ samples from the residential, industrial, and traffic sources were 0.99, 1.35, and 1.30, respectively. This indicated that the emission source in the residential area of Nanjing was dominated by stationary sources, while the industrial and traffic sources were dominated by mobile sources. OC and EC accounted for a large proportion of PM_2.5_, accounting for 7.35–15.30% of the PM_2.5_ mass concentration. The OC/EC ratio was often used to pinpoint PM_2.5_ secondary sources. The OC/EC ratios of the PM_2.5_ samples from three sampling points in Nanjing were mainly distributed between 6 and 8, indicating that PM_2.5_ in Nanjing was mainly secondary. The heavy metals accounted for 3.93%, 5.09%, and 5.76% of the PM_2.5_ samples from the residential, industrial, and traffic sources, respectively. The significant differences in the heavy metals content between particulate matter from different sources indicated a relationship with the pollution sources in their respective regions. In terms of PAHs, 16 types of PAHs (naphthalene was not detected) accounted for 0.00483%, 0.00612%, and 0.00913% of the mass concentration of PM_2.5_ from the residential, industrial, and traffic sources, respectively. The trend of the total concentration of PAHs was traffic source (91.31 ng/mg) > industrial source (61.15 ng/mg) > residential source (48.30 ng/mg). The content of PAHs varied significantly among different sources of particulate matter, indicating that the source of PM_2.5_ had an impact on the content of PAHs.

### 3.2. Cell Vitality

**Figure 2:**
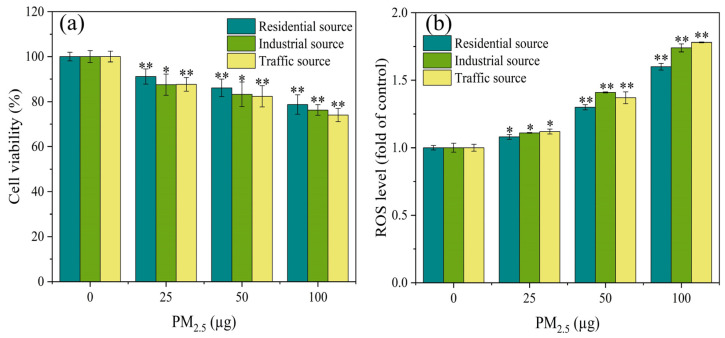
After 4 h of exposure at the ALI, (**a**) the cell viability and (**b**) ROS level of A549 cells were observed. Compared with the control group, “*” means *p* < 0.05, and “**” means *p* < 0.01.

As demonstrated in Figure 2a, the cell viability of the A549 cells exhibited significant differences (*p* < 0.05) from the control group at PM_2.5_ exposure levels of 25, 50, and 100 μg. Additionally, the exposure dose of PM_2.5_ was in a dose–response relationship with the cell viability, and the higher the exposure dose, the lower the cell viability. The A549 cells were exposed to PM_2.5_’s organic components in Nanjing’s industrial and urban districts, and the results showed that the inhibitory effect on the cell viability in the industrial area samples was greater than that in the urban area samples during winter and spring [31]. The results in this study were similar, with differences in the toxicity of particles from different sources on the A549 cells. The toxicity of PM_2.5_ from the traffic source was the strongest, followed by the industrial source, and the residential source had the lowest toxicity. This might be related to differences in the chemical composition of PM_2.5_ from different sources. The results of the component analysis indicated that water–soluble ions, PAHs, and heavy metals components had the highest content in the traffic–source samples, followed by the industrial areas, and the lowest content in the residential sources. This might be the reason for the differences in cytotoxicity of PM_2.5_ from the different sources.

### 3.3. ROS Generation

The production of ROS could damage cellular macromolecules, such as DNA, and proteins. The ROS level was an important indicator reflecting the degree of oxidative damage to organisms [32,33]. After 4 h of exposure at the ALI, the ROS level of the A549 cells was measured, as shown in Figure 2b. At low exposure dosages, all particulate matter exposure groups significantly increased the ROS concentration of the A549 cells in comparison to the control group (*p* < 0.05). At the PM_2.5_ exposure of 50 μg, the industrial source of PM_2.5_ induced higher levels of ROS in the cells compared to the residential and transportation sources. As the PM_2.5_ exposure dose increased, the ROS levels in each exposure group showed a trend of increasing, indicating that PM_2.5_ exposure induced oxidative stress in the cells. According to a study conducted in India, traffic PM_2.5_ particles had a greater degree of oxidative potential, produced more ROS, and caused more DNA damage and cell death in human respiratory cells than PM_2.5_ from different areas (rural, urban, and industrial) [23]. The results of this study also confirmed the previous report that, overall, after acute exposure, the traffic–source PM_2.5_ samples produced more ROS than the other samples. This difference in ROS generation might be attributed to the samples’ high concentration of heavy metals and PAHs.

### 3.4. Expression Level of Inflammatory Factors

**Figure 3:**
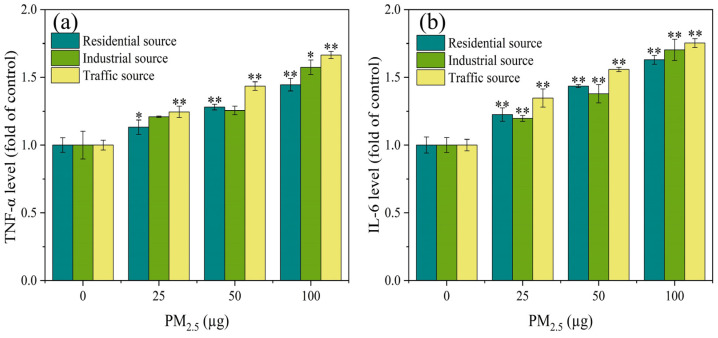
The expression levels of TNF–α (**a**) and IL–6 (**b**) in A549 cells after 4 h of ALI exposure. Compared with the control group, the expression levels of TNF–α and IL–6 in each exposure group were significantly increased, and there was a positive correlation between PM_2.5_ exposure levels, “*” means *p* < 0.05, and “**” means *p* < 0.01.

Relevant research has shown that oxidative stress in cells led to the increased expression of inflammatory factors, which in turn caused inflammatory damage to the body [29]. As shown in Figure 3a, compared with the control group, there was a significant difference in the expression level of TNF–α in particulate matters from the residential and traffic sources at PM_2.5_ exposure doses of 25 μg to 100 μg (*p* < 0.05). When the exposure level of PM_2.5_ was 100 μg, compared with the control group, the industrial particulate matters caused a significant increase in the TNF–α levels (*p* < 0.05). When the exposure dose of PM_2.5_ was 25–100 μg, there was a significant difference in the expression level of IL–6 between the exposure groups and the control group (Figure 3b) (*p* < 0.01). The expression level of IL-6 increased in a dose-dependent manner. At PM_2.5_ exposure levels of 25 and 50 μg, the impact of the industrial source of PM_2.5_ on IL–6 secretion was relatively low compared to the residential and traffic sources. The experimental results indicated that with the increase in PM_2.5_ exposure, the inflammatory damage to the cells became more severe, and there was a significant difference in the toxicity of the particulate matter sources. The impact of the traffic–source PM_2.5_ samples on the expression of inflammatory factors was higher than that of the residential and industrial sources. Some related studies have found that different seasons and sources of particulate matter had varying degrees of influence on the secretion of inflammatory factors (IL–8 and IL–6). The metals, PAHs, endotoxin, and other components in particulate matter played an important role in the inflammatory response induced by PM_2.5_ [34,35]. These results suggested that the source and spatiotemporal distribution of particulate matter could lead to different components of particulate matter, which in turn affected its biological toxicity.

### 3.5. Cell Apoptosis Analysis

**Figure 4:**
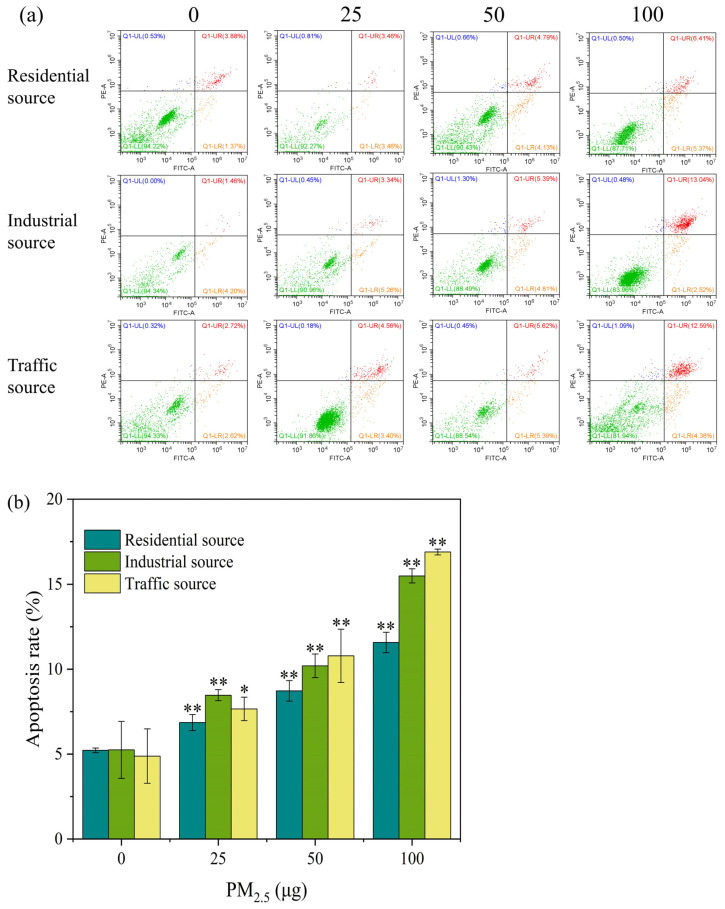
(**a**) Apoptosis flow cytometry of A549 cells exposed to PM_2.5_ at the ALI for 4 h. The exposure concentrations from left to right are 0, 25, 50, and 100 μg, respectively. Compared with the control group, the apoptosis rate of each exposure group increased in a dose–dependent manner. Green, orange, red, and blue within the quadrant represent the proportion of live cells, early apoptotic cells, late apoptotic cells, and dead/necrotic cells, respectively. (**b**) The apoptosis rate of A549 cells after 4 h of exposure. “*” means *p* < 0.05, “**” means *p* < 0.01.

PM_2.5_ exposure could cause necrosis and apoptosis in cells. The Annexin V–FITC/PI double–stained method was used to determine the apoptosis rate of the A549 cells after they were exposed to the ALI for 4 h. As shown in Figure 4a, with the increase in the exposure dose of particulate matter, the early apoptosis rate and the mid–late apoptosis rate of the A549 cells showed an upward trend. Except for the low–exposure group of the industrial particulate matter, the mid–late apoptosis rate was generally higher than the early apoptosis rate. In Figure 4b, compared with the control group, the apoptosis rates of the three exposed samples were significantly increased at a dose of 25 μg (*p* < 0.05), and the trend increased as the exposure dose increased. When the exposure level of particulate matter was 25 μg, the industrial–source PM_2.5_ caused more cell apoptosis, and when the exposure level of particulate matter was 50 and 100 μg, the traffic–source PM_2.5_ caused a higher degree of cell apoptosis. The above results indicated that acute exposure to PM_2.5_ could lead to cell apoptosis. It has been reported that short–term exposure to PM_2.5_ in Ningxia and Qinghai regions induced cell cycle arrest in A549 cells, led to apoptosis or necrosis, and damaged cells might not be able to repair [5]. In addition, the trend of changes in the apoptosis rate and cell viability were opposite, but the overall trend showed that the cytotoxicity of particulate matter from the traffic source was stronger, followed by the toxicity of the industrial source, and the toxicity of the residential source was lower.

### 3.6. DNA Damage

**Figure 5:**
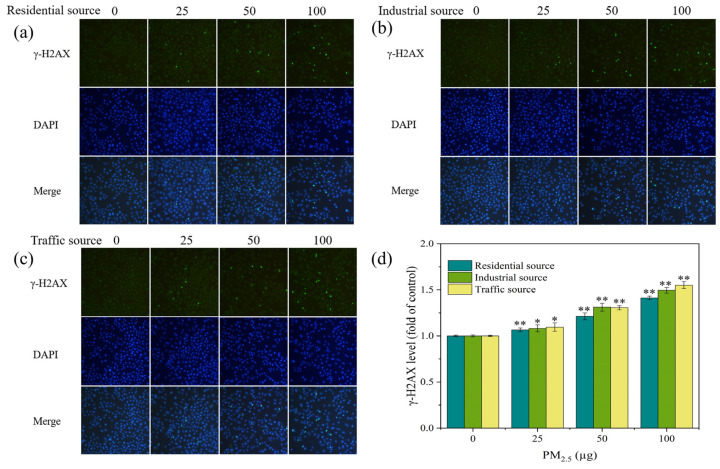
(**a**–**c**) Fluorescence images of γ–H2AX in A549 cells exposed to PM_2.5_ at the ALI for 4 h. The exposure concentrations from left to right are 0, 25, 50, and 100 μg, respectively. Compared with the control group (0 μg), the fluorescence intensity of each exposure group increased. (**d**) Level of γ–H2AX in A549 cells after 4 h of exposure. Compared with the control group, “*” means *p* < 0.05, and “**” means *p* < 0.01.

H2AX is a variant of the histone H2A family. A common biomarker of DNA double–strand breaks (DSBs) is γ–H2AX, which is produced when H2AX is phosphorylated. The level of γ–H2AX might indicate the extent of DNA damage [4,36]. In this experiment, stained γ–H2AX showed green fluorescence under a fluorescence microscope (Figure 5a–c). In each exposure group, there were more fluorescent spots and a higher fluorescence intensity as compared to the control group. As the exposure dose of the particulate matter increased, the number and intensity of fluorescent dots increased, indicating an increase in the γ–H2AX content and an aggravation of cellular DNA damage. By calculating the fluorescence intensity of each image, the DNA damage level of A549 cells was quantified, as shown in Figure 5d. At low exposure doses, each exposure group could significantly increase the levels of γ–H2AX in cells when compared to the control group (*p* < 0.05). As the PM_2.5_ exposure dose increased, the levels of γ–H2AX in cells significantly increased (*p* < 0.01), indicating an increase in the DNA damage levels. At PM_2.5_ exposure levels of 50 μg, the industrial–source samples caused relatively high levels of cellular DNA damage. A study has shown that the exposure of cells to fresh and aged smoke particles at the air–liquid interface could induce an increase in the DNA damage levels in cells, and the toxicity of aged smoke particles was stronger [37]. In this study, under acute exposure conditions at the air–liquid interface, the traffic–source PM_2.5_ induced the highest levels of γ–H2AX and DNA damage in cells, the industrial–source PM_2.5_ had a higher degree of DNA damage in cells, and the residential–source PM_2.5_ had the lowest impact on the DNA damage. The results suggested that the traffic, industrial, and residential PM_2.5_ had different chemical compositions, resulting in different levels of DNA damage. The trend of the DNA damage changes was consistent with the trend of the ROS level changes, which might be due to oxidative stress-induced DNA damage. It has been reported that PM_2.5_ caused DNA strand breakage through the production of ROS, affecting the genetic toxicity of cells [38,39,40]. ROS could interact with DNA molecules, inducing DNA strand breaks and causing oxidative DNA damage [41,42].

### 3.7. Correlation Analysis between Biological Toxicity Indicators and Chemical Components of PM_2.5_

According to earlier research, the chemical compositions of PM_2.5_ played a significant role in causing biological toxicity effects, and the toxicity effects produced by different components vary [43]. The correlation between the chemical makeup of PM_2.5_ and the cytotoxicity effects was analyzed (Table 1, Table 2, and Table S6). NH_4_^+^, Cl^−^, SO_4_^2−^, and NO_3_^−^ were highly negatively correlated with the cell viability. In addition to Na^+^, K^+^, and Mg^2+^, other water–soluble ions were highly correlated with ROS, TNF–α, IL–6, the apoptosis rate, and DNA damage. Carbon components (OC and EC) were highly correlated with toxicity indicators such as ROS, inflammatory factors, and DNA damage. PAHs and endotoxin were highly correlated with the cytotoxicity effect indicators. According to a study conducted in Jinan, PM_2.5_ and its chemical constituents could cause lung damage and generate cytotoxicity. The majority of inorganic elements (including Hg, Pb, and Cr) and PAHs had a strong negative correlation with the cell viability and were crucial for cytotoxicity [44]. For heavy metals, Al, Mn, Fe, As, Cd, Pb, Sb, and Ti were highly negatively correlated with the cell viability. In addition to Cr, Ni, Cu, Se, Ba, and Sr, most heavy metals were highly positively correlated with ROS, TNF–α, IL–6, the apoptosis rate, and DNA damage. Previous similar studies have found that the toxicity indicators were related to the content of metal elements such as Zn, Fe, Cr, Mn, Cd, Ni, As, Cs, and Pb in atmospheric particulate matter [17,45]. When exposed to PM_2.5_ in Nanjing’s urban and industrial districts, the industrial–source samples typically produced more cytotoxicity than the urban–source samples [31]. This indicated that there were differences in the particulate matter toxicity in different regions, which was generally consistent with the results of this study. The overall trend of cytotoxicity produced by the PM_2.5_ samples was traffic–source samples > industrial–source samples > residential–source samples. This might be related to differences in the chemical composition of PM_2.5_. The results of the component analysis indicated that water–soluble ions, PAHs, and heavy metals components had the highest content in the traffic–source samples, followed by the industrial–source samples, and the residential–source samples had the lowest content. According to the Pearson correlation analysis (Table 1 and Table 2), several cytotoxicity effect indicators were highly correlated with PAHs, endotoxin, carbon components, and heavy metals components in the PM_2.5_ samples, and there was a significant correlation between the cytotoxicity indicators and several water–soluble ions. The water–soluble ions, carbon components, PAHs, endotoxin, and heavy metals in PM_2.5_ might be the main elements that caused cytotoxicity, according to our analysis of the aforementioned results.

The hypothesis that oxidative stress was the initial step in the toxic effects induced by fine particulate matter has been widely accepted [46,47]. Some research results supported oxidative stress as an important mechanism for PM_2.5_–induced inflammatory response, cytotoxicity, and carcinogenesis. Wang et al. [48]. investigated the mechanism of particulate matter–induced lung inflammation and found that particulate matter exposure led to the activation of ROS–mediated MAPK kinases (ERK, JNK, p38MAPK) and the downstream NF–κB signaling pathway, which in turn induced airway inflammation. This suggested that oxidative stress played a crucial role in particulate matter–induced pulmonary inflammation. Yang et al. [5]. found that human lung cancer cells exposed to particulate matter could lead to oxidative stress and apoptosis. This study showed that acute exposure to PM_2.5_ at the ALI significantly raised the generation of ROS, suggesting that PM_2.5_ caused oxidative stress in cells under acute exposure. The formation of ROS and the presence of PAHs and heavy metals in PM_2.5_ were significantly correlated, according to the Pearson correlation analysis. This implied that oxidative stress caused by PM_2.5_ might be significantly influenced by PAHs and heavy metals. Numerous investigations have also verified that ROS production could be triggered by heavy metals and PAHs [20,49]. Furthermore, OC and water–soluble ions had a strong correlation with the generation of ROS. The fact that water–soluble ions could break down into cells and stimulate ROS generation might be a significant contributing factor [50,51]. Water–soluble ions make up the majority of the PM_2.5_ samples, according to the component analysis of the samples, and they might be involved in inducing the production of ROS in cells. According to the Pearson correlation analysis between the toxicity effect indicators (Supporting Information, Table S7), ROS generation was strongly correlated with the cell survival rate, TNF–α, IL–6, cell apoptosis rate, and DNA damage. This suggested that ROS–mediated oxidative stress was an important mechanism for the PM_2.5_–induced toxicity effects, which was also consistent with the results of other studies [5,40,52]. 

## 4. Conclusions

This study explored the toxic effects on A549 cells of PM_2.5_ from distinct regions in Nanjing. The research results found that acute exposure to particle matter from different sources at the air–liquid interface caused damage to cells, led to apoptosis, oxidative stress, inflammatory damage, and DNA damage. The important potential mechanism of PM_2.5_–induced biological toxicity was related to oxidative stress. The health hazards of the PM_2.5_ samples from the traffic source were higher than those from the industrial and residential sources, due to differences in the chemical composition. Water–soluble ions, carbon components, PAHs, endotoxin, and heavy metals were key components that caused cytotoxicity. The findings of this study contributed to understanding the impact of living in urban environments on human health and provided a reference for evaluating the relevant toxic components of PM_2.5_.

## Figures and Tables

**Table 1:** Pearson correlation coefficient between cytotoxic effect indexes and main chemical components of PM_2.5_. “**” means *p* < 0.01.

	Cell Viability	ROS	TNF–α	IL–6	Apoptosis Rate	DNA Damage
Na^+^	–0.646	0.716	0.730	0.771	0.632	0.718
NH_4_^+^	–0.858 **	0.983 **	0.946 **	0.928 **	0.977 **	0.973 **
K^+^	–0.575	0.673	0.538	0.631	0.506	0.636
Mg^2+^	–0.662	0.757	0.677	0.755	0.612	0.734
Ca^2+^	–0.781	0.876 **	0.880 **	0.898 **	0.822 **	0.876 **
F^−^	–0.788	0.885 **	0.887 **	0.903 **	0.831 **	0.884 **
Cl^−^	–0.863 **	0.982 **	0.962 **	0.949 **	0.969 **	0.977 **
SO_4_^2−^	–0.862 **	0.984 **	0.943 **	0.947 **	0.944 **	0.973 **
NO_3_^−^	–0.858 **	0.981 **	0.951 **	0.929 **	0.980 **	0.973 **
OC	–0.721	0.807 **	0.803 **	0.841 **	0.725	0.805 **
EC	–0.778	0.872 **	0.875 **	0.894 **	0.814 **	0.872 **
PAHs	–0.829 **	0.935 **	0.957 **	0.921 **	0.959 **	0.941 **
Endotoxin	–0.824 **	0.939 **	0.937 **	0.890 **	0.978 **	0.939 **

**Table 2:** Pearson correlation coefficient between cytotoxic effect indexes and heavy metals in PM_2.5_. “**” means *p* < 0.01.

	Cell Viability	ROS	TNF–α	IL–6	Apoptosis Rate	DNA Damage
Al	–0.828 **	0.958 **	0.870 **	0.888 **	0.896 **	0.934 **
V	–0.798	0.929 **	0.826 **	0.845 **	0.866 **	0.902 **
Cr	–0.646	0.718	0.722	0.769	0.624	0.717
Mn	–0.829 **	0.960 **	0.873 **	0.888 **	0.903 **	0.937 **
Fe	–0.823 **	0.950 **	0.864 **	0.893 **	0.872 **	0.927 **
Co	–0.789	0.918 **	0.810 **	0.844 **	0.832 **	0.889 **
Ni	–0.651	0.773	0.621	0.672	0.664	0.732
Cu	–0.327	0.374	0.284	0.401	0.183	0.348
Zn	–0.731	0.847 **	**0.736**	0.800 **	0.718	0.816 **
As	–0.857 **	0.983 **	0.939 **	0.924 **	0.971 **	0.972 **
Se	–0.690	0.797	0.692	0.766	0.655	0.768
Cd	–0.810 **	0.941 **	0.847 **	0.857 **	0.891 **	0.916 **
Ba	–0.513	0.589	0.493	0.594	0.414	0.562
Pb	–0.809 **	0.939 **	0.839 **	0.865 **	0.865 **	0.912 **
Sr	–0.473	0.535	0.471	0.569	0.368	0.516
Sb	–0.828 **	0.959 **	0.883 **	0.878 **	0.931 **	0.939 **
Ti	–0.827 **	0.930 **	0.952 **	0.928 **	0.937 **	0.936 **

## Data Availability

Data are contained within the article and Supplementary Materials.

## Supplementary Materials

The following supporting information can be downloaded at: https://www.mdpi.com/article/10.3390/toxics12010021/s1, Table S1: Content of water–soluble ions in PM_2.5_. Table S2: Content of OC and EC in PM_2.5_. Table S3: Concentrations of metal elements in PM_2.5_ as measured by ICP–MS. Table S4: Content of PAHs in PM_2.5_ obtained from GC–MS. Table S5: Content of endotoxin in PM_2.5_. Table S6: Pearson correlation coefficient between cytotoxic effect indexes and PAHs of PM_2.5_. Table S7: Pearson correlation coefficient between cytotoxic effect indexes in A549 cells.
