# Supplementary material for: The Cytotoxic Effects of Fine Particulate Matter (PM2.5) from Different Sources at the Air–Liquid Interface Exposure on A549 Cells
Source: Toxics. 2023 Dec 25;12(1):21. doi: 10.3390/toxics12010021 (PMC10821317; doi:10.3390/toxics12010021)
Supplement: Supplementary file 1 [file toxics-12-00021-s001.zip › toxics-2767595-supplementary.pdf]

Table S1

Content of water−soluble ions in $PM_{2.5}$.

Ions	Residential−source Mean(mg/g)	Industrial−source Mean(mg/g)	Traffic−source Mean(mg/g)
Na^{+}	1.29	0.40	1.14
NH_4^{+}	10.44	15.25	14.65
K^{+}	1.53	1.04	0.47
Mg^{2+}	0.30	0.16	0.16
Ca^{2+}	2.06	1.26	2.20
F^{-}	0.17	0.11	0.18
Cl^{-}	2.98	3.50	3.93
SO_4^{2-}	21.06	22.75	23.63
NO_3^{-}	21.17	31.07	30.97

Table S2

Content of OC and EC in $PM_{2.5}$.

Species	Residential−source Mean ± SD (μg/m^3)	Industrial−source Mean ± SD (μg/m^3)	Traffic−source Mean ± SD (μg/m^3)
OC	6.07 ± 2.48	7.05 ± 1.87	10.37 ± 4.65
EC	0.76 ± 0.66	1.14 ± 0.45	1.52 ± 0.81

Table S3

Concentrations of metal elements in $PM_{2.5}$ as measured by ICP−MS.

Metal element	Residential−source Mean(mg/g)	Industrial−source Mean(mg/g)	Traffic−source Mean(mg/g)
Al	5.26	6.99	5.60
V	0.30	0.46	0.34
Cr	1.01	0.39	1.19
Mn	2.52	3.36	2.60
Fe	12.34	15.39	14.43
Co	0.02	0.03	0.02
Ni	0.08	0.22	0.17
Cu	0.87	0.99	5.09
Zn	12.20	17.41	21.13
As	0.58	0.61	0.42
Se	0.17	0.23	0.33
Cd	0.10	0.14	0.10
Ba	0.62	0.73	1.79
Pb	2.14	3.07	2.53
Sr	0.60	0.38	1.51
Sb	0.24	0.32	0.20
Ti	0.24	0.15	0.15

Table S4

Content of PAHs in $PM_{2.5}$ obtained from GC−MS.

Species	Residential−source Mean(ng/mg)	Industrial−source Mean(ng/mg)	Traffic−source Mean(ng/mg)
Naphthalene	0.21	0.39	0.22
Acenapthene	0.29	0.68	0.62
Acenapthylene	0.45	0.52	0.87
Fluorene	0.54	0.90	1.14
Phenanthrene	2.69	3.75	6.46
Anthracene	0.77	0.35	1.83
Fluoranthene	7.52	7.81	13.86
Pyrene	7.16	9.33	13.35
Benzo[a]anthracene	2.78	4.89	5.77
Chrysene	4.48	5.02	9.36
Benzo[b&k]fluoranthene	6.33	7.34	11.28
Benzo[a]pyrene	2.79	3.83	5.55
Indenopyrene	5.40	7.10	9.15
Benzo[ghi]perylene	5.48	7.60	9.58
Dibenzo[ah]anthracene	1.40	1.65	2.29

Table S5

Content of endotoxin in $PM_{2.5}$.

Species	Residential−source Mean ± SD (EU/mg)	Industrial−source Mean ± SD (EU/mg)	Traffic−source Mean ± SD (EU/mg)
Endotoxin	0.06 ± 0.01	0.11 ± 0.01	0.12 ± 0.01

Table S6

Pearson correlation coefficient between cytotoxic effect indexes and PAHs of $PM_{2.5}$.

"**" means $p < 0.01$.

Species	Cell viability	ROS	TNF−α	IL−6	Apoptosis rate	DNA damage
Naphthalene	-0.762	**0.893****	0.791	0.789	**0.862****	**0.866****
Acenapthene	**-0.809****	**0.930****	**0.900****	**0.857****	**0.963****	**0.923****
Acenapthylene	**-0.812****	**0.912****	**0.947****	**0.910****	**0.939****	**0.921****
Fluorene	**-0.831****	**0.942****	**0.951****	**0.907****	**0.978****	**0.945****
Phenanthrene	-0.788	**0.884****	**0.930****	**0.880****	**0.933****	**0.896****
Anthracene	-0.623	0.677	0.780	0.739	0.721	0.704
Fluoranthene	**-0.807****	**0.904****	**0.941****	**0.909****	**0.925****	**0.914****
Pyrene	**-0.835****	**0.942****	**0.959****	**0.924****	**0.965****	**0.947****
Benzo[a]anthracene	**-0.832****	**0.947****	**0.947****	**0.903****	**0.981****	**0.947****

Chrysene	-0.790	**0.883****	**0.931****	**0.890****	**0.919****	**0.896****
Benzo[b&k]fluoranthene	**-0.830****	**0.935****	**0.957****	**0.926****	**0.952****	**0.941****
Benzo[a]pyrene	**-0.828****	**0.934****	**0.955****	**0.915****	**0.964****	**0.940****
Indenopyrene	**-0.850****	**0.964****	**0.965****	**0.936****	**0.975****	**0.964****
Benzo[ghi]perylene	**-0.849****	**0.963****	**0.963****	**0.932****	**0.978****	**0.963****
Dibenzo[ah]anthracene	**-0.847****	**0.958****	**0.964****	**0.939****	**0.964****	**0.959****

Table S7

Pearson correlation coefficient between cytotoxic effect indexes in A549 cells.

"**" means $p < 0.01$.

	cell viability	ROS	TNF−α	IL−6	apoptosis rate	DNA damage
Cell viability	1	**-0.847****	**-0.860****	**-0.853****	**-0.824****	**-0.848****
ROS	**-0.847****	1	**0.927****	**0.923****	**0.955****	**0.987****
TNF-α	**-0.860****	**0.927****	1	**0.953****	**0.950****	**0.928****
IL-6	**-0.853****	**0.923****	**0.953****	1	**0.897****	**0.927****
apoptosis rate	**-0.824****	**0.955****	**0.950****	**0.897****	1	**0.947****
DNA damage	**-0.848****	**0.987****	**0.928****	**0.927****	**0.947****	1
